# Experimental and analytical study on the performance of an efficient octagonal greenhouse solar chimney as a solar dryer

**DOI:** 10.1038/s41598-026-57043-x

**Published:** 2026-06-16

**Authors:** Aya Gamal, I. T. Zedan, Ahmed M. Elsayed, H. H. El-Ghetany

**Affiliations:** 1 Renewable Energy Science and Engineering Department, Faculty of postgraduate studies for advanced sciences, Beni-Suef University, Beni-Suef, 62511 Egypt; 2 Mechanical Engineering Department, Fayoum University, Fayoum, 63514 Egypt; 3 Solar Energy Department, National Research Centre, Dokki, 12622 Giza Egypt

**Keywords:** Solar dryer, Solar radiation, Moisture content, Performance evaluation, Octagonal greenhouse, Energy science and technology, Engineering, Environmental sciences

## Abstract

Agricultural product drying is one of the most widely used preservation techniques for reducing post-harvest losses and extending shelf life. However, conventional solar dryers often suffer from non uniform airflow distribution, thermal instability, airflow stagnation zones, and limited drying efficiency under varying climatic conditions. To address these limitations, this study experimentally and analytically investigates the performance of a novel octagonal greenhouse solar dryer integrated with a solar chimney under different seasonal operating conditions. Unlike conventional rectangular and trapezoidal configurations commonly reported in the literature, the proposed octagonal geometry was designed to enhance airflow circulation, improve thermal distribution uniformity, minimize stagnant airflow regions, and provide improved structural stability under wind loading conditions. Four representative months (March, June, September, and December) were selected to evaluate seasonal performance and simulate year-round operating conditions. Both leafy products (Molokhia and mint) and non-leafy products (tomato and onion) were experimentally and analytically examined under different climatic conditions. The results showed that the proposed system effectively utilized available solar energy, achieving an average thermal efficiency of approximately 50%. The required solar energy to reach the target moisture content was approximately 7.8, 8.1, 4.4, and 3.35 kWh/m² for tomato, onion, Molokhia, and mint, respectively. Leafy products exhibited lower energy demand and were successfully dried within one day, while tomato required approximately 1.5–2 days and onion required 2–3 days to reach safe moisture levels .Thermal analysis indicated a progressive increase in air temperature from ambient conditions to absorber plate temperatures, with the highest performance observed in June and the lowest in December. Furthermore, experimental validation conducted on 15 March showed strong agreement between predicted and measured results, confirming the reliability of the proposed analytical model. Overall, the proposed octagonal greenhouse solar chimney dryer exhibits improved thermo-aerodynamic performance compared with conventional greenhouse dryer geometries and provides a reliable low-energy solution for agricultural drying applications.

## Introduction

 Drying food is one of the oldest methods of food preservation and remains highly relevant today. Previous studies indicates that while water content is essential for the growth of bacteria, yeasts, and fungi, dried food is resistant to their growth. According to these studies, dried agricultural products have several advantage over fresh ones, the most important being an extended shelf life, which enables the availability of nutrients from off season products throughout the year. Additionally, dried products are easier to handle, package, and store than fresh foods, making them suitable for transportation from production areas to distant markets^1^.

Dehydration or drying involves the removal of water content from fruits or vegetables until the desired moisture content is reached. Studies have demonstrated that water removal can be achieved using varies physical techniques, such as solar drying^2^.

Agricultural drying is widely recognized as an energy-intensive process despite its extensive industrial applications. Previous studies have identified that the main controlling parameters during drying are the temperature of the drying air and the relative humidity of the surrounding environment^3^.

However, while these studies agree on the importance of these parameters, most of them primarily focus on process conditions without fully addressing system level energy parametric analysis.

In addition, air recirculation has been proposed as an effective strategy for reducing relative humidity and improving drying efficiency^4^.

Nevertheless, this approach is often evaluated in isolation, without comprehensive integration with other energy-saving mechanisms or system configurations.

Similarly, conventional drying systems are generally reported to rely heavily on energy consumption for air circulation, heating, and dehumidification processes to achieve acceptable drying performance. From an economic perspective, previous investigations have highlighted that operational costs particularly energy, labor, and initial capital play a dominant role in the overall cost structure of drying systems^5^.

For example, it has been reported that energy consumption may account for more than 30% of total drying costs, while initial capital investment can reach approximately 46% under specific price conditions^5^. Although this study clearly demonstrates the economic burden of energy use, it does not explore design-level solutions to fundamentally reduce energy demand. Consequently, many researchers have focused on improving energy efficiency in drying systems^6–9^.

Among these efforts, renewable energy based solutions, particularly solar drying and natural ventilation systems, have been widely investigated. While solar drying has been shown to significantly reduce energy costs compared to conventional electrical drying methods, most studies primarily emphasize cost reduction and product quality improvement^10,11^, with limited attention given to integrated system design parametric analysis and performance enhancement under varying environmental conditions.

Overall, the literature indicates that although significant progress has been made in understanding drying parameters, energy consumption, and solar-based solutions, there remains a lack of comprehensive studies that simultaneously integrate thermal performance, airflow behavior, system design parametric analysis, and economic efficiency within a unified framework. Solar dryers are generally classified into direct and indirect systems, each exhibiting distinct performance characteristics. Direct solar dryers are often reported to achieve relatively higher thermal efficiencies ranging from 15% to 40% depending on climatic conditions, dryer configuration, and product type^12–15^.

However, despite this advantage, indirect systems have been shown to better preserve product quality, including vitamins and color, due to the absence of direct solar radiation exposure^12^. These findings indicate a trade off between thermal efficiency and product quality that remains a key design challenge in solar drying systems.Several experimental and numerical studies have further demonstrated that variations in solar radiation significantly influence heat transfer coefficients, collector efficiency, and overall drying rates^16–25^.

Nevertheless, most of these investigations primarily focus on system performance under varying environmental conditions, with limited attention given to integrated design parametric analysis. In addition, airflow in most solar drying systems is commonly assisted using fans or blowers^25^, which introduces additional energy demand and partially offsets the benefits of solar energy utilization. Regarding operating conditions, it has been observed that increasing drying air temperature at constant air velocity can reduce total energy consumption due to shorter drying time^26^.

In contrast, other studies have reported that operating at lower temperatures increases energy demand due to prolonged drying duration, while higher air velocities result in increased energy consumption^27^.

Furthermore, experimental evidence shows that increasing air velocity from 1.0 m/s to 1.5 m/s and 2.5 m/s leads to a nearly proportional increase in energy consumption. These contradictory findings highlight the complex interaction between airflow, temperature, and energy efficiency, and indicate that optimal operating conditions are highly system dependent rather than universally defined^28,29^.

From a technological perspective, different drying methods exhibit significant variation in energy consumption. For instance, microwave drying has been reported to consume approximately 70% less specific energy compared to conventional drying techniques^30^. However, despite its efficiency, conventional solar drying remains more environmentally sustainable and economically accessible, particularly when integrated with greenhouse structures and thermal energy storage systems^31^.

In this article, solar drying has been widely recognized as a promising solution for reducing post-harvest losses, which account for approximately 30–40% of global food production, especially in developing countries^32^.

Although traditional open sun drying is cost-effective, it is associated with several limitations including poor hygiene, long drying times, and high product quality degradation. Consequently, recent reviews have emphasized the importance of integrating renewable energy systems, thermal storage, and hybrid configurations to improve energy efficiency, product quality, and environmental performance^32^. However, many of these approaches still rely on empirical or trial-and-error design methodologies, which limit their predictive capability and parametric analysis potential. In this regard, recent studies have proposed performance-based analytical approaches for solar dryer design, enabling improved collector design, optimized airflow pathways, and enhanced thermal performance^33,34^.

Mathematical modeling has also been widely adopted to predict system behavior under varying conditions, with the aim of reducing energy consumption, cost, and drying time^35^. Nevertheless, the integration of rigorous analytical modeling with experimental validation remains limited, despite its potential to provide a more reliable and scalable framework for system parametric analysis.

This integration represents a key methodological advancement that underpins the present study. Recent advancements in solar chimney systems have further demonstrated their potential to enhance airflow and overall system performance when integrated with drying applications. For example, CFD based investigations have shown that design modifications such as stepped absorbers can significantly improve pressure distribution and airflow velocity, with some configurations achieving up to 39 times higher power output due to enhanced mixing and heat transfer^36^.

Similarly, hybrid systems combining solar chimneys with different turbine types and absorber materials have demonstrated improved efficiency, highlighting the importance of both material selection and system integration^37^.

Moreover, parametric analysis studies involving more than 40 configurations have confirmed that geometric factors such as chimney angle, collector height, and shape significantly influence performance, with airflow velocity improvements of up to 200% reported under optimized conditions^38^.

Despite these advancements, existing studies largely focus on either thermal performance, geometric parametric analysis, or system integration in isolation. A comprehensive approach that simultaneously combines geometric parametric analysis, airflow enhancement, thermal analysis, and experimental validation within a unified framework remains limited in the literature. This gap forms the basis for the present study, which investigates an integrated octagonal greenhouse solar chimney system using both experimental and analytical methodologies.

Previous studies have investigated various solar dryer geometries, including rectangular, triangular, and trapezoidal configurations, to enhance airflow distribution and improve drying performance^39^. However, despite these efforts, these conventional designs still exhibit inherent limitations, particularly in terms of non-uniform air circulation, thermal gradients, and the formation of stagnant airflow zones, which ultimately reduce overall drying efficiency. Similarly, other studies have examined the effect of different dryer geometries on thermal behavior and drying performance by comparing commonly used configurations such as rectangular, triangular, and trapezoidal designs in terms of airflow distribution, temperature uniformity, and thermal efficiency^40^.

Elsayed et al.^41^ investigated the enhancement of Solar Chimney Power Plant performance by integrating wavy geometries into the collector roof. Different wave configurations were analyzed using CFD simulations to evaluate their effects on airflow behavior, pressure distribution, and temperature enhancement. The results demonstrated that optimized wavy designs can significantly improve airflow rate and thermal performance, offering an effective passive approach for increasing the efficiency of sustainable energy systems.

Elsayed et al.^42^ illustrated the performance enhancement strategies for Solar Chimney Power Plant by introducing curved guide vanes inside the collector to address spatial limitations and improve energy output without increasing system size. The proposed design modifies the airflow pattern by inducing a controlled swirl motion, which extends the air path within the collector, enhances heat transfer, and increases the kinetic energy available at the chimney entrance. A detailed numerical analysis was conducted using CFD simulations to evaluate different configurations of guide vanes, along with variations in collector diameter and vane number. The results showed that optimized configurations significantly improved system performance, with notable increases in average inlet velocity, reductions in pressure drop, and enhanced turbine efficiency. The best performance was achieved with eight guide vanes, leading to substantial gains in power output and overall efficiency. The study concludes that incorporating curved guide vanes offers a promising and scalable passive technique to improve the aerodynamic and thermal performance of solar chimney systems under various operating conditions.

Cuce et al.^43^ investigated the integration of industrial waste heat into a Solar Chimney Power Plant to enable continuous power generation even during low or no solar radiation conditions. Using a 3D CFD model based on the Manzanares pilot plant, the system performance was analyzed under solar radiation of 400 W/m² and ambient temperature of 294 K, combined with waste heat at 816.9 K from a gas power plant. The results showed that incorporating waste heat significantly enhanced system performance, increasing the power output from 20.68 kW to 33.807 kW (an improvement of 63.47%). Additionally, the system was able to generate 14.016 kW during periods with no sunlight, demonstrating its potential for 24 h electricity production.

The previous studied investigated the thermal behavior, drying kinetics, and economic feasibility of solar dried cocoyam chips using a specially designed solar dryer with a partitioned collector. The modified design increases airflow path length, promotes turbulence, and enhances heat and mass transfer during drying. The system was tested under humid harvesting conditions, and results showed that drying took about 25 h to reach equilibrium under variable weather conditions.

Gupta et al.^44^ revealed that airflow velocity ranged between laminar and turbulent regimes, while thermal properties such as specific heat capacity, thermal conductivity, and thermal effusivity decreased with moisture content, whereas thermal diffusivity increased. Among the tested models, the two-term model best described the drying behavior. Economically, the solar dryer demonstrated strong potential for cost savings and short payback periods, confirming its efficiency and practical applicability for agricultural drying processes.

Gupta et al.^45^ focused on improving a PV-assisted solar dryer as a clean and sustainable alternative to traditional energy-intensive drying methods. The main idea was to enhance heat transfer inside the dryer by modifying the absorber surface using square obstacles with threaded pin fins, which increase turbulence and improve energy absorption. Two designs were tested: a conventional absorber and a modified one, under different airflow rates. The results showed that the modified design significantly improved drying performance, reducing moisture content more effectively and achieving higher drying, thermal, and exergy efficiencies. It also provided economic benefits through a short payback period and substantial CO2 reduction. Overall, the study demonstrates that optimizing absorber design can greatly enhance the performance and sustainability of solar drying systems for food preservation.

The findings consistently indicate that dryer geometry plays a crucial role in internal air circulation and heat transfer characteristics. Conventional geometries such as triangular, trapezoidal, rectangular, and hexagonal configurations often suffer from airflow non-uniformity and localized stagnation regions due to uneven internal flow paths and asymmetrical air distribution. Although geometric modifications may partially improve airflow behavior, these issues are not completely eliminated in most traditional designs.

In contrast, the proposed octagonal geometry demonstrated superior performance owing to its enhanced symmetry and balanced internal structure, which facilitate more uniform airflow circulation and minimize stagnation zones^46^. As a result, the octagonal dryer achieved improved thermal distribution and more stable drying conditions compared with previously reported geometries. Furthermore, the integration of an experimental–analytical framework combined with annual-scale solar radiation assessment provides a more comprehensive evaluation of system performance and strengthens the practical applicability of the proposed design beyond conventional geometry based studies^47^.

The present study proposes an octagonal solar powered vegetable drying system that extends previous investigations on conventional geometries such as triangular, hexagonal, and other standard configurations. Unlike these commonly studied designs, the proposed octagonal configuration introduces improved geometric symmetry, which contributes to enhanced airflow uniformity and more stable thermal behavior within the drying chamber. The study provides several key contributions. First, it quantifies the solar radiation dose (kWh/m²/day) required for drying both leafy and non leafy agricultural products under different seasonal conditions throughout the year, addressing a parameter that has received limited attention in the existing literature. Second, the work integrates experimental measurements with theoretical modeling under identical operating conditions, enabling accurate prediction of absorber plate and chamber temperature distributions. This combined experimental analytical framework improves the reliability of performance evaluation and enhances the predictive capability of the proposed system. Overall, the proposed approach offers a more comprehensive assessment of solar drying performance by combining geometric parametric analysis, seasonal solar analysis, and validated modeling within a unified framework.

A performance-based analytical parametric analysis approach is employed to enhance airflow distribution, collector efficiency, and overall thermal performance of the proposed solar drying system. This methodology enables a more systematic evaluation of design and operating parameters compared to conventional empirical approaches. In addition, the study demonstrates the feasibility of constructing the dryer using low cost, locally available materials, supporting its potential scalability and suitability for rural and off grid applications. This contributes to improving accessibility of sustainable drying technologies in resource-limited settings. By integrating seasonal and product specific performance analysis with combined experimental and theoretical validation, along with the proposed octagonal design configuration, the study provides a comprehensive framework for evaluating and optimizing solar drying systems in terms of energy efficiency and operational performance.

The octagonal geometry in solar dryers provides a favorable design configuration due to its ability to enhance solar radiation interception from multiple orientations, which can potentially improve overall energy absorption. Its geometric similarity to a circular contributes to reducing thermal losses and promoting a more uniform temperature distribution within the drying chamber. In addition, this configuration is expected to improve internal airflow patterns by enhancing flow circulation and reducing stagnant zones, which can positively influence heat and mass transfer processes and overall drying performance compared to conventional geometries. The drying experiments were conducted under nature convection conditions, where a fan installed at the chimney inlet was used to enhance airflow through the system. This assisted ventilation improves air movement within the drying chamber, thereby supporting more effective heat and mass transfer during the drying process.

## Mathematical modeling of solar dryer

The mathematical model of the solar dryer is developed based on fundamental principles of thermodynamics and heat transfer. The formulation is derived by applying the conservation of energy principle to the drying chamber, where the incoming solar energy is balanced with the useful energy gained by the airflow and the thermal losses from the system. The model assumes a steady-state operation, uniform thermo physical properties of air, and one-dimensional airflow through the drying chamber. Based on these physical principles, the energy balance equations are formulated to describe the heat transfer processes within the system, including convective heat gain by the air stream and the energy required for moisture evaporation from the agricultural products. Efficient heat transfer, uniform temperature distribution and the effective moisture removal are depend on the thermal performance on the dryer. Efficient heat transfer, uniform temperature distribution, and effective moisture removal are directly governed by the thermal performance of the drying system. In this study, the main performance indicators, including heat losses, spatial temperature distribution, and overall thermal efficiency, are systematically analyzed to evaluate system behavior. The findings demonstrate that maintaining a stable and uniform air temperature significantly enhances drying kinetics, reduces processing time, and improves the quality of the dried products.

Although simplified assumptions were adopted in the development of the mathematical model, they are commonly used in solar drying and heat transfer studies to reduce mathematical complexity while maintaining acceptable engineering accuracy. These assumptions are considered valid within the investigated operating range and do not significantly affect the overall prediction of system performance.

This expression is derived by applying the first law of thermodynamics to the control volume of the dryer. The heat energy balance of the solar dryer can be calculated expressed as follows^48^:

1 $$\:{\:Q}_{T}={Q}_{U}+{Q}_{Loss}$$

Where $\:{\:\:Q}_{T}$ is the total solar heat energy available for the dryer (W), $\:{Q}_{U}$is the useful heat gain (W) and Q_Loss_ represents the total heat losses from the system (W). The useful heat gain representing the convective heat transfer between the air stream and the drying chamber can be calculated as:

2 $$\:{Q}_{U}=\dot{{m}_{a}}{C}_{p}({T}_{out}-{T}_{in})$$

where $\:\dot{{m}_{a}}$ is the mass flow rate of air (kg/s), *C*_*p*_ is the specific heat capacity of air (J/kg.K), $\:{T}_{out}$ is the outlet air temperature (°C), and $\:{T}_{in}$ is the inlet air temperature (°C).

The temperature difference ΔT ($\:{T}_{out}-{T}_{in}$) represents the temperature differential between the collector’s outflow air and the ambient surrounding air.^49,50^.The mass of water removed during the drying process was calculated using Eq. (3)^49^.

3 $$\:{M}_{w}=\frac{{m}_{i}-{m}_{f}}{100-{m}_{f}}\times\:W$$

Where $\:{\:\:m}_{i}$ is the initial moisture content (%), $\:{m}_{f\:}$is the final moisture content (%), W is the total weight of the fresh product (kg), and $\:{M}_{w}$ is the mass of water removed during drying (kg).

The total energy required for moisture removal was calculated using Eq. (4) which accounts for both sensible and latent heat requirements during the drying process

4 $$\:Q=W{C}_{p}\left({T}_{d}-{T}_{a}\right)+{M}_{w}\lambda\:$$

Where Q is a total energy required (kJ),$\:{\:T}_{d}$ is the drying temperature (^ᴼ^C), $\:{T}_{a}\:$ is the ambient temperature (^ᴼ^C), and $\:\lambda\:$ is Latent heat of vaporization (kJ/kg).

The required solar collector area, $\:{A}_{c}$ was determined using Equation^51^.

5 $$\:{A}_{c}=\frac{\dot{m}{C}_{p}\left({T}_{d}-{T}_{a}\right)}{{\eta\:}_{c}{I}_{g}}$$

Where $\:\dot{m}$ is the mass flow rate of air (kg/s) and $\:{I}_{g}$ is the daily solar radiation (kWh/m^2^).

6 $$\:\dot{m}={\rho\:}_{air}{V}_{ch}{A}_{ch}$$

Where $\:{V}_{ch}$ is air velocity in chimney and $\:{\rho\:}_{air}$ is air density (kg/m^3^).

The drying efficiency $\:{\eta\:}_{d}$ of the solar dryer was calculated using equation^52^:

7 $$\:{\eta\:}_{d=\:\frac{{m}_{w\:{L}_{v}}}{I\:{A}_{t}}}$$

where $\:{m}_{w}$ is the mass of moisture removed (kg), $\:{L}_{v}$ is the latent heat of vaporization (kJ/kg), I is the solar radiation intensity (W/m²), A is the drying area (m²), and t is the drying time (s). The calculated drying efficiency was found to be approximately 50%, indicating a reasonable utilization of solar energy for moisture removal under the studied operating conditions. This value falls within the typical range reported for solar drying systems. Tomato and onion samples were prepared as uniform slices with thicknesses of approximately 5 mm and 4–5 mm, respectively, to ensure consistent drying conditions and experimental reproducibility.

The chosen material’s initial moisture content crops such as tomato, onion, mint, and Molokhia presented in Table 1. And some assumption used for calculation the moisture ratio is shown in Table 2.

**Table 1:** Initial moisture content (wet basis, %) of selected agricultural products^53–55^.

Product	Moisture content Initial (%)
Tomato	95
Onion	80
Molokhia	87
Mint	69

**Table 2:** Information and presumptions utilized in the moisture content calculation.

Parameters	value
Mass of products (kg)	50
Latent heat capacity of water (kJ/kg)	2257
Tomato Thickness	5 mm
Onion Samples	4–5 mm

The calculated drying efficiency was found to be approximately 50%, indicating a reasonable utilization of solar energy for moisture removal under the studied operating conditions. This value falls within the typical range reported for solar drying systems.

### Governing assumptions

Steady-state conditions, uniform air properties, negligible heat losses (if applicable), one-dimensional airflow assumption, constant latent heat value and uniform initial moisture distribution.

## Experimental setup

**Fig. 1:**
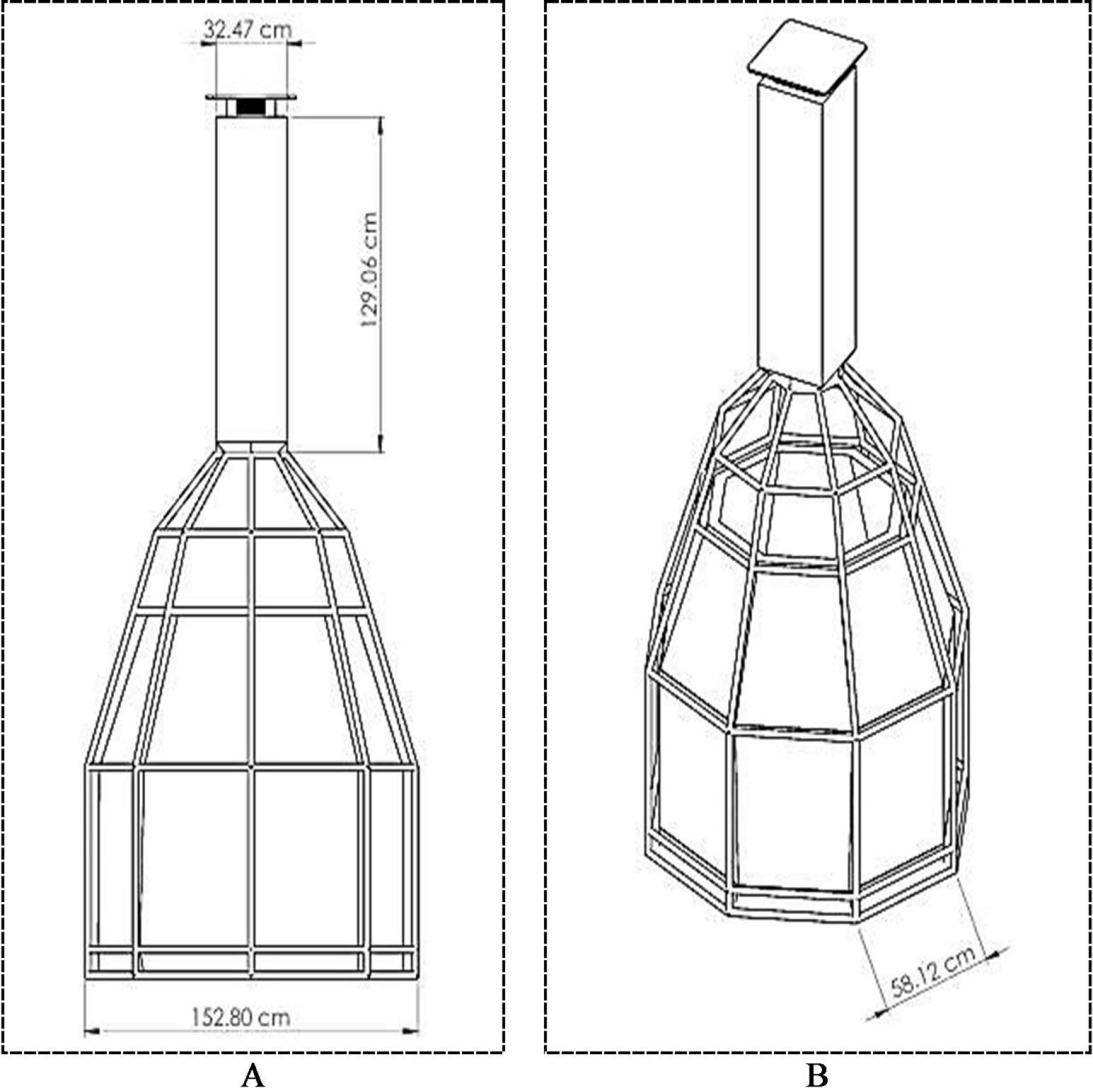
**A** 2D and **B** 3D views of octagonal system geometry with overall structural dimensions.

The solar drying system was designed, constructed, and tested under the prevailing meteorological conditions at the National Research Centre, Dokki, Giza, Egypt. The system consists of an integrated greenhouse type solar dryer with an octagonal geometry, as shown in Fig. 1.

### Geometrical configuration

The dryer is based on an octagonal base structure with a frontal width of approximately 152.80 cm and a side depth of 58.12 cm. The system transitions vertically into a converging upper section forming a truncated pyramidal chimney like shape with octagonal symmetry. This upper section has a height of 129.06 cm and terminates with an outlet opening of 32.47 cm. This gradual reduction in cross sectional area promotes airflow acceleration and enhances natural convection-driven air movement through the system.

### Structural design and materials

The structure is fabricated using an octagonal iron frame that provides mechanical stability and load bearing support. All external surfaces are covered with black steel panels (galvanized steel sheets) to improve solar absorption and ensure durability under prolonged exposure to environmental conditions. A metallic absorber plate is installed above the base to enhance heat retention and internal thermal stability. The entire system is covered with a transparent plastic sheet to facilitate solar radiation transmission and enhance the greenhouse effect within the drying chamber. The material properties of the solar dryer absorber plate sheet metal coated with black painted for absorptivity, emissivity, and thermal conductivity are 93%, 25%, 60 (W/m K) respectively. In the current system the insulating materials are not used. While the transmissivity of the transparent cover material was 85%.

### Sealing and insulation

All joints and structural connections are properly sealed to minimize air leakage and prevent dust ingress, which could affect the performance of the absorber surface and internal airflow behavior.

### System integration and components

**Fig. 2:**
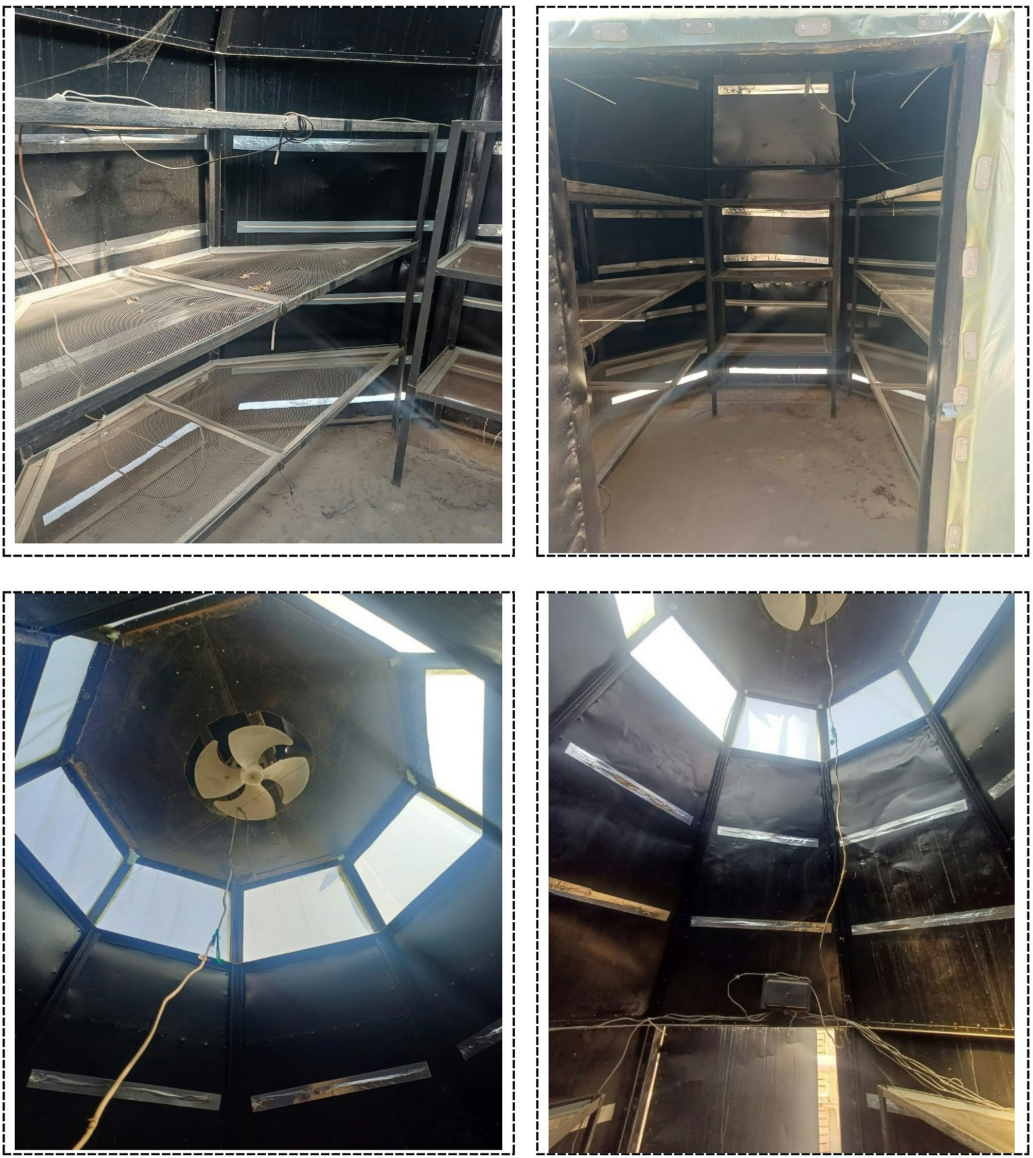
Detailed view of the system during assembly and component integration.

As illustrated in Fig. 2, the system includes internal shelving for product placement, a turbine generator unit, a belt mechanism, and a data storage system based on a memory card unit.

### Experimental repetition

Each drying experiment was conducted once under identical operating conditions to ensure repeatability and consistency of the results.

### Experimental procedure

**Fig. 3:**
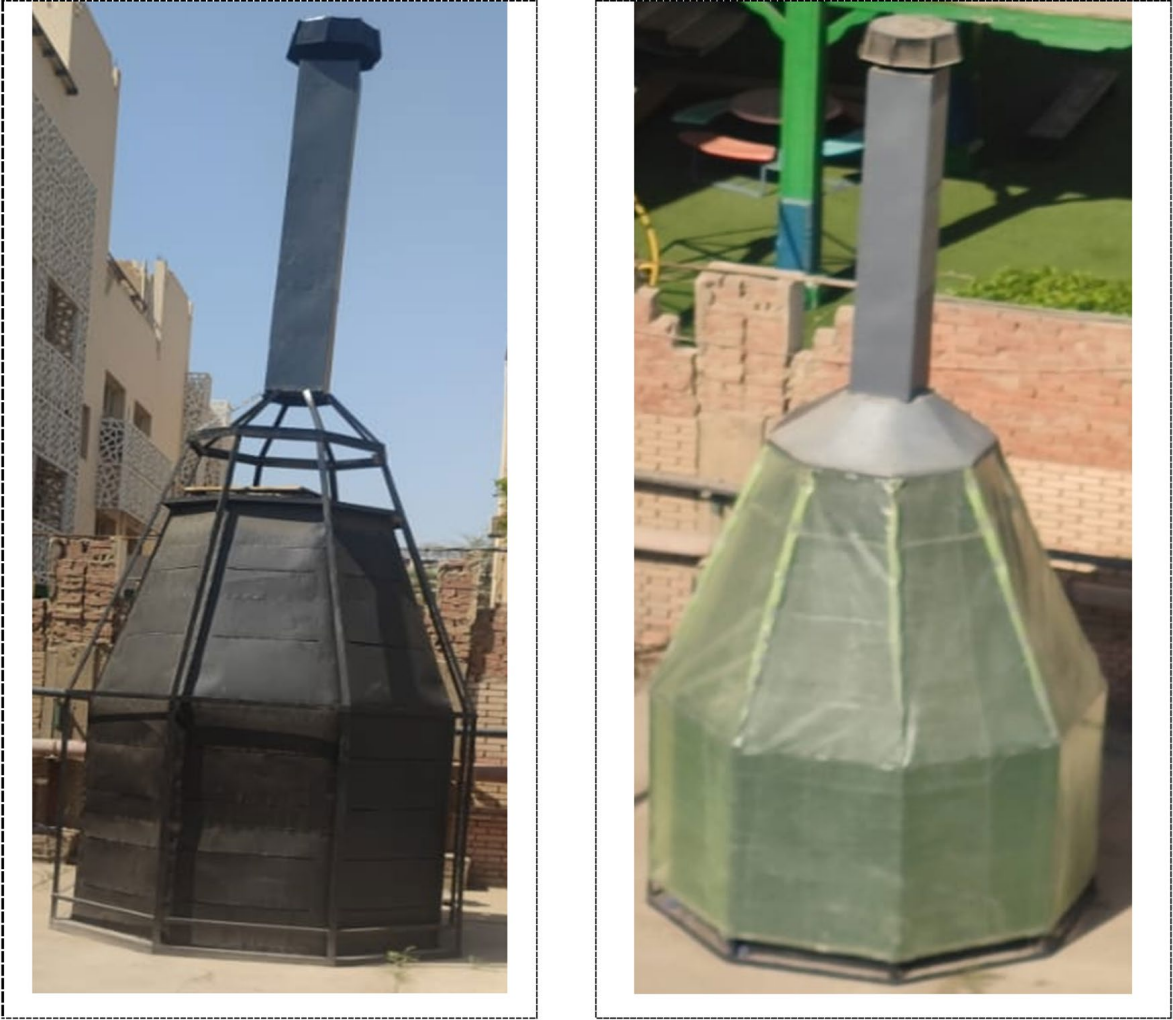
Photographic view of the built- in octagonal shape solar dryer.

**Fig. 4:**
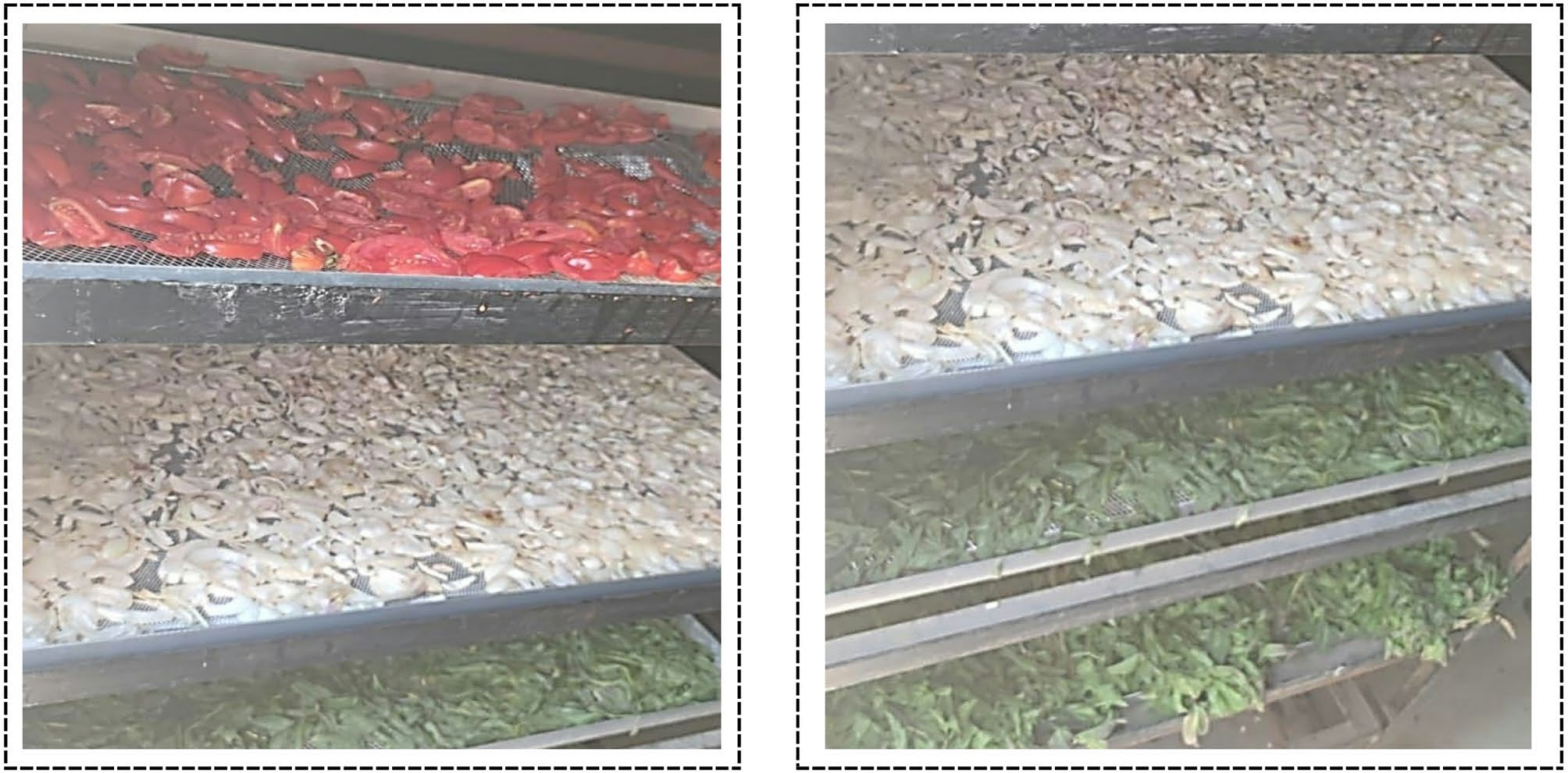
Fresh products placed on the internal dryer shelves.

Figure 3 illustrates the internal absorber surfaces and the external configuration of the octagonal solar dryer, while Fig. 4 shows the placement of fresh agricultural products on the internal drying shelves. During each experimental run, the system was first operated under no load conditions until steady state thermal behavior was reached. Temperature distribution inside the dryer was measured using Type T thermocouples installed at different locations within the drying chamber to capture spatial thermal variations. In addition, hourly measurements were recorded for ambient air temperature, relative humidity of inlet and outlet air streams, and solar radiation intensity. Fresh agricultural products were prepared prior to drying experiments. Onions and tomatoes were cut into thin slices using a stainless-steel knife, ensuring uniform thickness and consistent slicing orientation. All samples were then evenly distributed in a single layer on aluminum mesh shelves. The perforated structure of the shelves allowed uniform airflow circulation around and through the products, enhancing heat and mass transfer during drying. Different agricultural products, including tomato, onion, and leafy crops such as Molokhia and mint, were tested under identical operating conditions to evaluate system performance for varying moisture contents and product characteristics. The drying process was considered complete when the sample mass reached a constant value over successive measurements, indicating that equilibrium moisture content had been achieved. Moisture content was calculated and expressed on a wet basis (wb) throughout the study. The experimental drying chamber was designed with an overall height of approximately 140 cm and a tray section height of about 100 cm. Three drying trays were installed inside the chamber with an average spacing of 25 cm between consecutive trays to ensure uniform airflow and heat distribution during the drying process. Each tray had an approximate width of 90 cm, while the upper air outlet opening was about 17 cm.

## Measurements and instruments

The temperature distribution within the drying chamber was monitored using Type T thermocouples installed at multiple locations to capture spatial variations in thermal behavior. Ambient air temperature was also recorded continuously throughout the experimental runs. The mass of fresh and dried agricultural products was measured using a digital balance with a resolution of ± 1 g. Moisture content variation was determined using a digital moisture content meter to evaluate drying progress over time. Total solar radiation incident on the dryer surface was measured using a calibrated solar pyranometer. All measurements were recorded at regular time intervals during each experimental run to ensure consistency and reliability of the collected data.

### Instrument specifications and uncertainty analysis

The accuracy and uncertainty of all measuring instruments used in this study were evaluated to ensure the reliability and validity of the experimental data. Temperature measurements were conducted using Type T thermocouples installed at different locations within the drying chamber to capture spatial and temporal temperature variations. Solar radiation intensity was measured using a calibrated pyranometer. The mass of agricultural products during drying experiments was determined using a digital weighing balance with a resolution of ± 1 g. Moisture content was evaluated based on mass loss measurements using the same weighing device; therefore, its uncertainty is included within the mass measurement uncertainty. The uncertainty analysis for the measured variables was performed following the standard propagation of uncertainty method described by Holman [56]. The combined standard uncertainty was evaluated for solar radiation, air temperature, and product mass using the general formulation. The governing equations for the combined standard uncertainty *u(i)* for solar radiation measured by a digital pyranometer, air temperature *u(T)* measured by a digital temperature indicator using thermocouples, and product weight *u(w)* measured by a digital weighing balance meter are the general propagation of uncertainty formula shown in the following Eqs [56].

8 $$\:u\left(I\right)=\sqrt{\sum\:_{i=1}^{n}{\left(\frac{\partial\:I}{\partial\:{x}_{i}}\:u\left(xi\right)\right)}^{2}}$$

9 $$\:u\left(T\right)=\sqrt{\sum\:_{i=1}^{n}{\left(\frac{\partial\:T}{\partial\:{x}_{i}}\:u\left(xi\right)\right)}^{2}}$$

10 $$\:u\left(m\right)=\sqrt{\sum\:_{i=1}^{n}{\left(\frac{\partial\:m}{\partial\:{x}_{i}}\:u\left(xi\right)\right)}^{2}}$$

It is found that the maximum uncertainties of the solar radiation (pyranometer), air temperature (thermocouples), and product weight (weighing balance) were evaluated to be 2.0%, 2.25% and 1.5%, respectively. While the product moisture content was calculated from the measured product weight by digital weighing balance meter.

## Results and discussion

Four agricultural products, namely mint, onion, tomato, and Molokhia, were selected to evaluate the performance of the proposed solar drying system under different operating conditions. The experimental dataset was collected over multiple days and seasonal conditions (March, June, September, and December), representing different climatic scenarios to ensure a representative range of solar radiation and ambient temperature variations.

The system performance was evaluated based on the internal temperature distribution and the required solar radiation to achieve the final moisture content of the dried products. The temperature field within the solar dryer was recorded over different days and orientations to capture the temporal and spatial variations in thermal behavior.

**Fig. 5:**
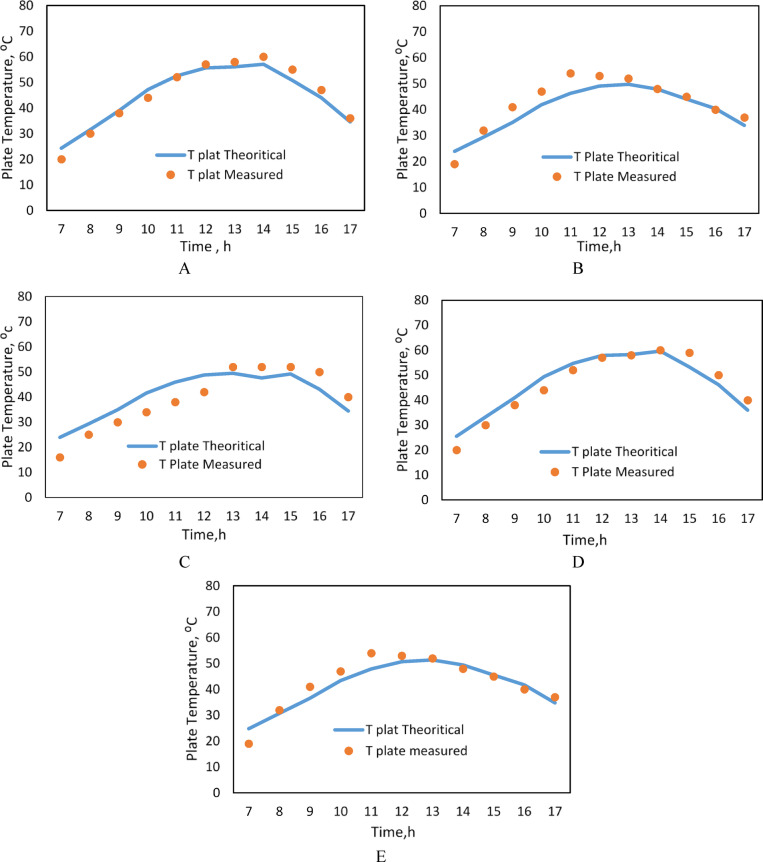
The Temperature distribution inside the solar dryer of drying experimental for different days in different directions **A** at 1 Mar South direction **B** at 1 Mar East Direction **C** at 1-Mar West Direction **D** at 15 Mar South Direction **E** at 15 Mar East Direction.

**Fig. 6:**
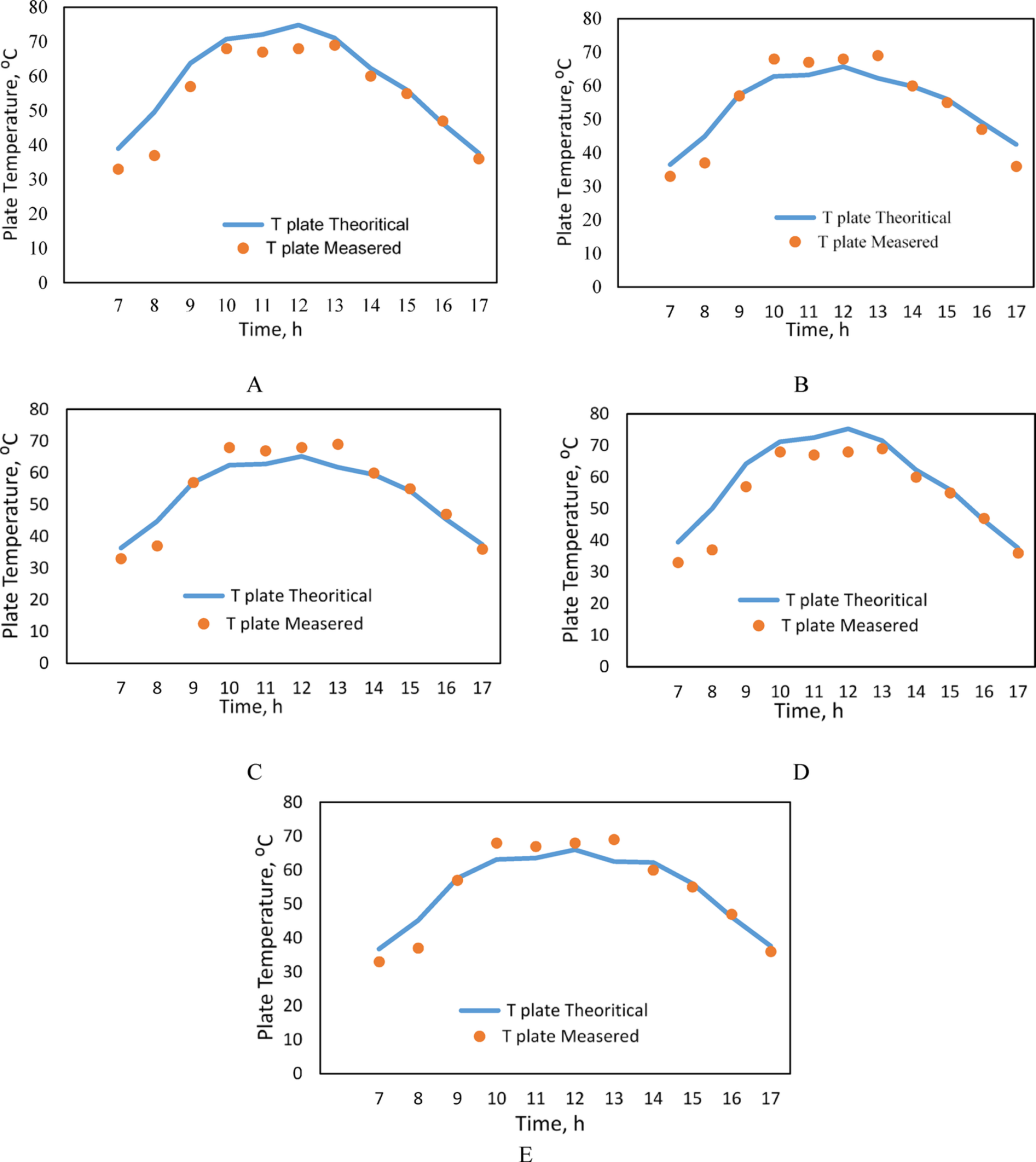
The Temperature distribution inside the solar dryer of drying experimental for different days in different directions **A** at 1 June South direction **B** at 1June East Direction **C**at June West Direction **D** at 15 June South Direction **E** at 1 June East Direction.

A comparison between theoretical predictions and experimentally measured plate temperatures is presented in Fig. 5. The results show a strong agreement between both datasets, with similar temporal trends and a consistent approach toward quasi-steady-state conditions. This close correspondence confirms the validity of the developed thermal model, while minor deviations are attributed to transient environmental fluctuations and measurement uncertainties. Figure 6 represents the results show a strong agreement between both datasets, with similar temporal trends and a consistent approach toward quasi-steady-state conditions. This close correspondence confirms the validity of the developed thermal model, while minor deviations are attributed to transient environmental fluctuations and measurement uncertainties. To further evaluate system behavior under varying climatic and operational conditions, temperature distributions for June were analyzed under different solar radiation incidence orientations on the system surfaces. This provides a more comprehensive understanding of the effect of solar angle variation on the thermal response and heat transfer characteristics within the drying chamber.

It is also important to clarify that the experimental dataset is not limited to March and June only, as measurements were additionally conducted for September and December under comparable operating conditions. These seasonal datasets confirm that the observed behavior is consistent across different climatic scenarios. However, March and June were selected as representative cases for detailed presentation, as they illustrate system performance under moderate and relatively high solar radiation conditions, respectively, while the same trends remain valid for September and December. In June, the variation of absorber plate temperature under different solar radiation orientations shows a strong agreement between the theoretical predictions and the experimental measurements. Both datasets follow a similar temporal trend, where the plate temperature increases during peak solar hours and gradually decreases toward the end of the day, reflecting the variation in solar intensity.

A clear convergence between the theoretical and measured values is observed across all considered orientations, indicating that the developed thermal model is capable of accurately capturing the dominant heat transfer mechanisms within the system under high solar radiation conditions. Although minor deviations are present, they remain within an acceptable range and can be attributed to transient fluctuations in solar radiation, heat losses to the surroundings, and simplifying assumptions in the model such as uniform airflow and steady state conditions.

Overall, the results confirm the validity and reliability of the proposed thermal model in predicting the absorber plate temperature behavior under varying solar incidence angles during June.

**Fig. 7:**
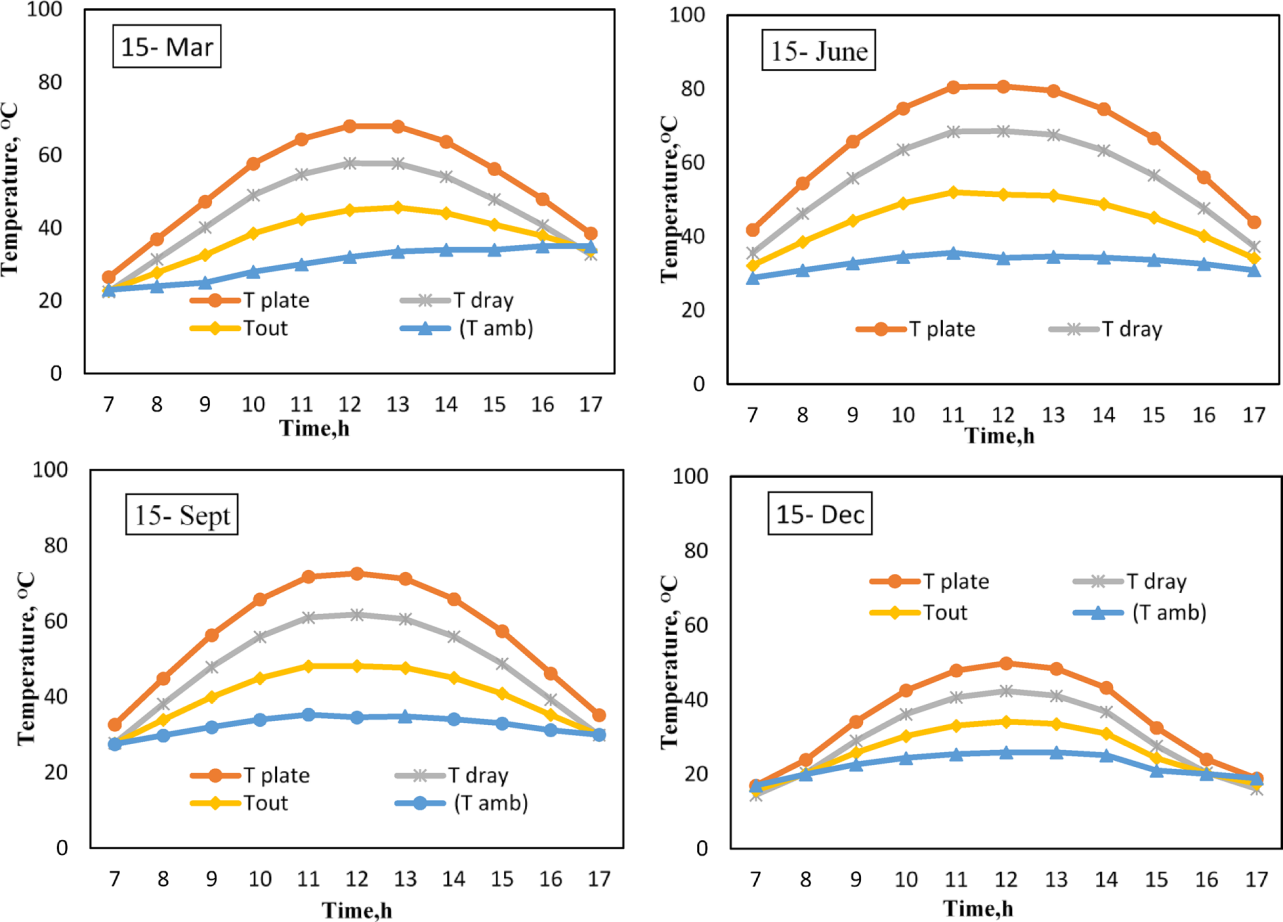
Calculated data of temperatures inside the solar dryer at the same day of different seasons.

To further evaluate system behavior under varying climatic conditions, temperature distributions were analyzed for representative days in each selected month (March, June, September, and December), as shown in Fig. 7. The results confirm a consistent thermal hierarchy (plate temperature > drying air temperature > outlet air temperature > ambient temperature), which aligns with established thermodynamic behavior in indirect solar drying systems.

Seasonal analysis indicates that June recorded the highest temperature levels due to increased solar radiation intensity and longer daylight duration, whereas December exhibited the lowest values due to reduced solar availability. These variations indicate that system performance is sensitive to dynamic weather conditions, which are not fully captured in the idealized analytical formulation. The recorded temperature ranges across the dataset demonstrate consistent and repeatable system behavior under different environmental conditions, supporting the adequacy of the dataset for model validation and performance assessment. The observed minor deviations between experimental and theoretical results can be attributed to transient environmental variations, fluctuations in solar radiation intensity, and simplifying assumptions in the thermal model such as steady-state conditions and uniform airflow distribution.

**Fig. 8:**
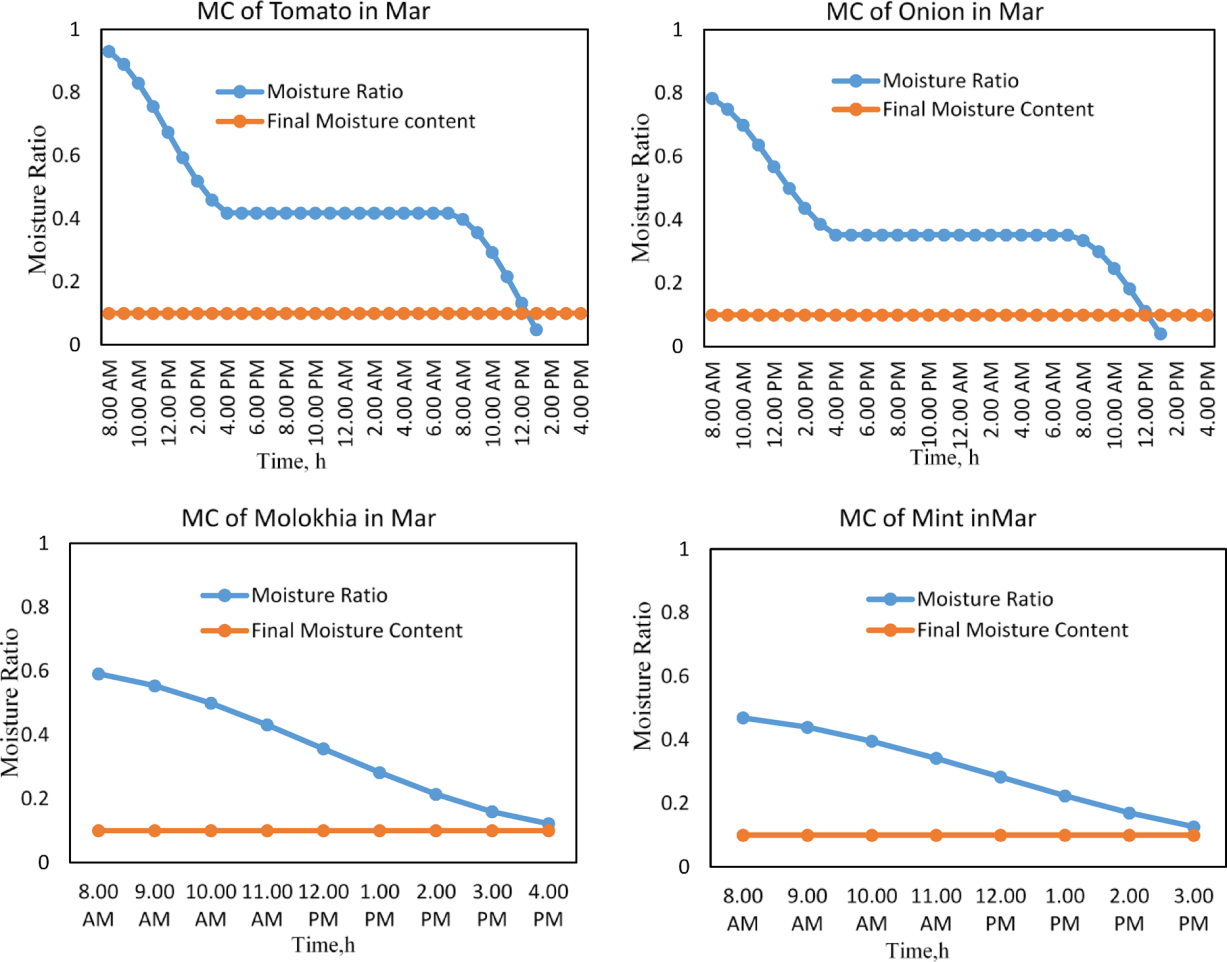
Moisture Content of different products in spring season.

Moisture content was calculated on a dry basis throughout the study to ensure consistency in data representation and facilitate comparison with previous drying studies. Figure 8 illustrates the variation of moisture content and moisture ratio of different agricultural products during solar drying in March. The moisture ratio was calculated using the standard drying model to describe the time-dependent reduction in moisture during the process.

For tomato slices, the initial moisture ratio was high due to the elevated initial water content. It decreased progressively during drying, reaching approximately 0.41 after about 8 h, and further declined to a final range of 0.05–0.07 after approximately 32 h, indicating that equilibrium conditions were approached. In contrast, onion slices exhibited a lower initial moisture content and a faster drying rate, with the moisture ratio decreasing to approximately 0.35 within the first 2 h and reaching a nearly constant value after about 26 h, indicating the end of the drying process.

The differences in drying behavior between tomato and onion can be attributed to variations in structural composition, porosity, and initial moisture content. Tomato, with its higher moisture content and denser cellular structure, required a longer drying time compared to onion, which facilitated faster moisture diffusion and evaporation.

Overall, the drying curves exhibited a typical two-stage behavior characterized by an initial rapid moisture removal phase followed by a slower falling rate period until the final drying condition was reached. The horizontal reference line at 0.1 represents the target final moisture ratio rather than a time-dependent measured value.

**Fig. 9:**
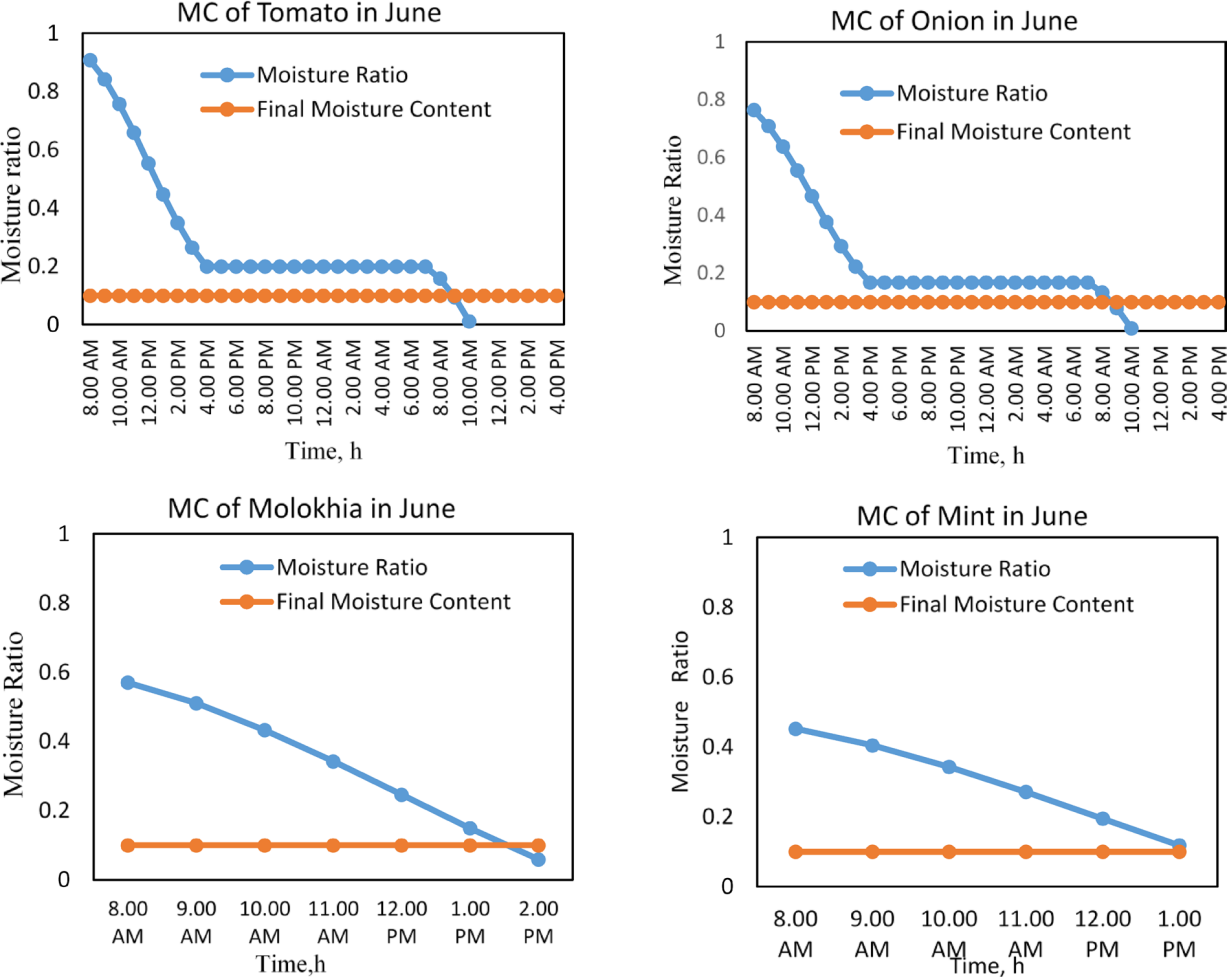
Moisture Content of different products in summer season.

In contrast to sliced products, leafy vegetables exhibited a faster moisture removal rate. For Molokhia leaves, the initial moisture content was approximately 59%, which decreased to about 12% within one full drying day (from 8:00 am to 4:00 pm), indicating rapid moisture diffusion due to their thin structure and high surface-area-to-volume ratio. Similarly, mint leaves showed an initial moisture content of about 46%, which decreased to nearly 12% within a single drying day (from 8:00 am to 3:00 pm), confirming the relatively fast drying behavior of leafy materials. Figure 9 illustrates the drying rate behavior of the tested agricultural products in June, showing a noticeable reduction in total drying time compared to March. This improvement is mainly attributed to higher solar radiation intensity and elevated drying temperatures, which enhance heat and mass transfer rates within the drying chamber. For tomato slices, the total drying time was reduced to approximately one day plus the initial 1–2 h of the following day to reach the final moisture content. Onion slices exhibited a similar trend but with a shorter overall drying duration compared to March, indicating improved drying kinetics under higher thermal conditions. However, leafy vegetables demonstrated significantly faster drying rates. Molokhia leaves reached their final moisture content by approximately 2:00 pm, while mint leaves completed drying earlier at around 1:00 pm. This rapid drying behavior is attributed to their porous structure, lower thickness, and reduced internal resistance to moisture migration. The observed results confirm that increasing drying temperature in June significantly enhances moisture removal rates for all tested materials. This behavior is consistent with the principles of heat and mass transfer, where higher thermal energy increases vapor pressure gradients and accelerates moisture diffusion from the product to the surrounding air. Furthermore, the results agree with previous studies on solar drying systems, which reported that higher ambient temperatures and solar intensities lead to shorter drying times and improved drying efficiency, particularly for thin and leafy agricultural products.

**Fig. 10:**
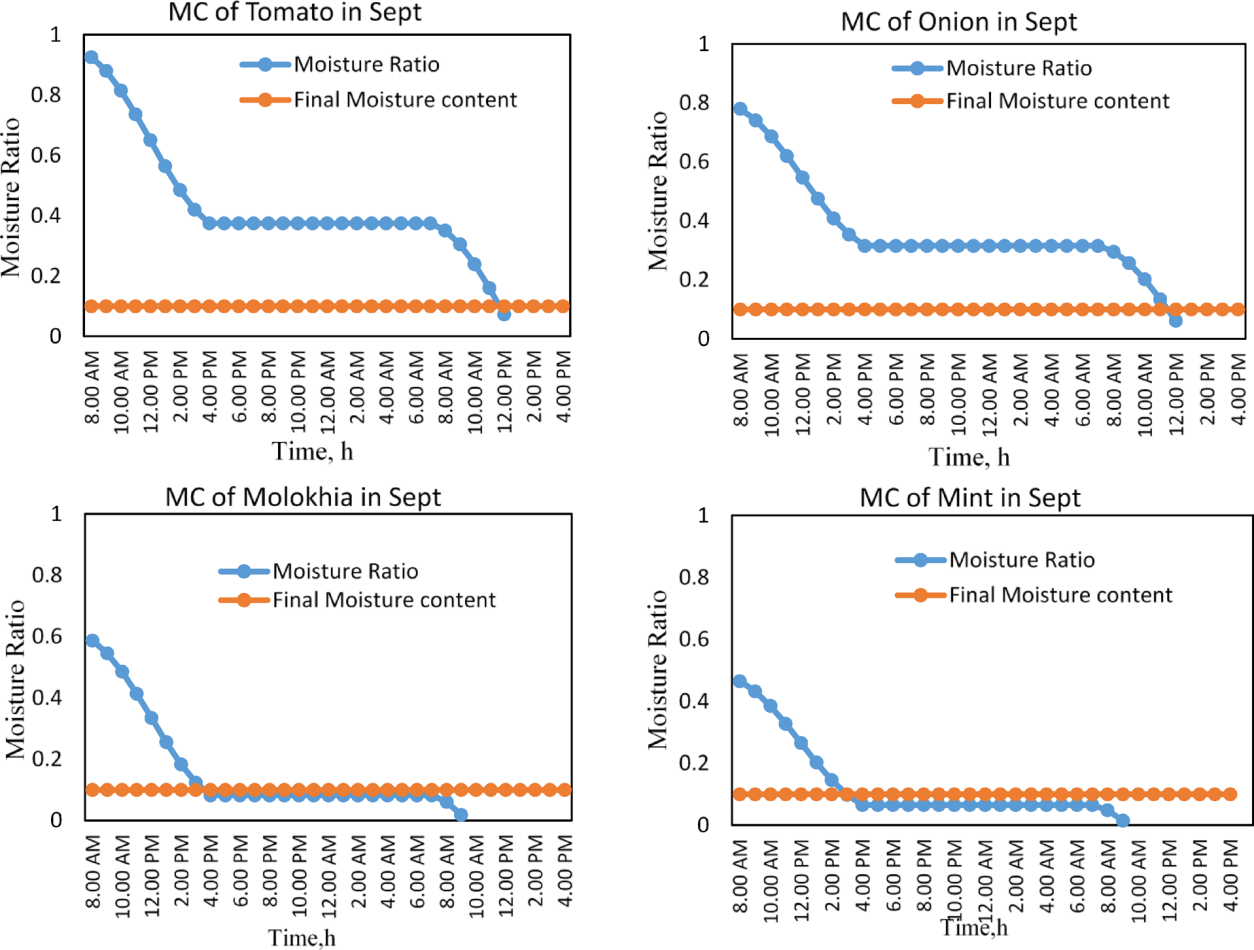
Moisture Content of different products in autumn season.

Figure 10 presents the variation of moisture content and moisture ratio of different agricultural products during the drying process in September. The results indicate a consistent decrease in moisture content over time for all tested products, confirming the effectiveness of the solar drying system under moderate seasonal conditions. For tomato slices, the initial moisture ratio was approximately 0.91, which decreased to about 0.37 after 8 h of drying. The value further declined to a final range of 0.05–0.07 after approximately 32 h of drying, indicating that equilibrium moisture content had been achieved. In the case of onion slices, the initial moisture ratio was approximately 0.77, decreasing to about 0.30 after 2 h from the beginning of the drying process. The drying process continued until the next day, reaching a nearly constant value after approximately 29–30 h, indicating the end of significant moisture removal. For leafy vegetables, a faster drying behavior was observed. Molokhia leaves, with an initial moisture content of approximately 58%, decreased to about 18% within one drying day (8:00 AM to 4:00 PM), and reached the final moisture level after approximately two additional hours on the second day. Similarly, mint leaves showed an initial moisture content of about 45%, which decreased rapidly to near equilibrium within one drying day, followed by a short stabilization period on the second day. Figure 10 illustrates the daily variation of moisture content for all products in December. The results show a slower drying rate compared to September, which can be attributed to lower solar radiation intensity and reduced ambient temperature during winter conditions. For tomato slices, the moisture content decreased from approximately 94% at 8:00 AM to about 63% by 4:00 PM on the first drying day. On the second day, it further decreased from 63% to 32%, followed by a gradual approach to equilibrium moisture content with only minor changes thereafter.

**Fig. 11:**
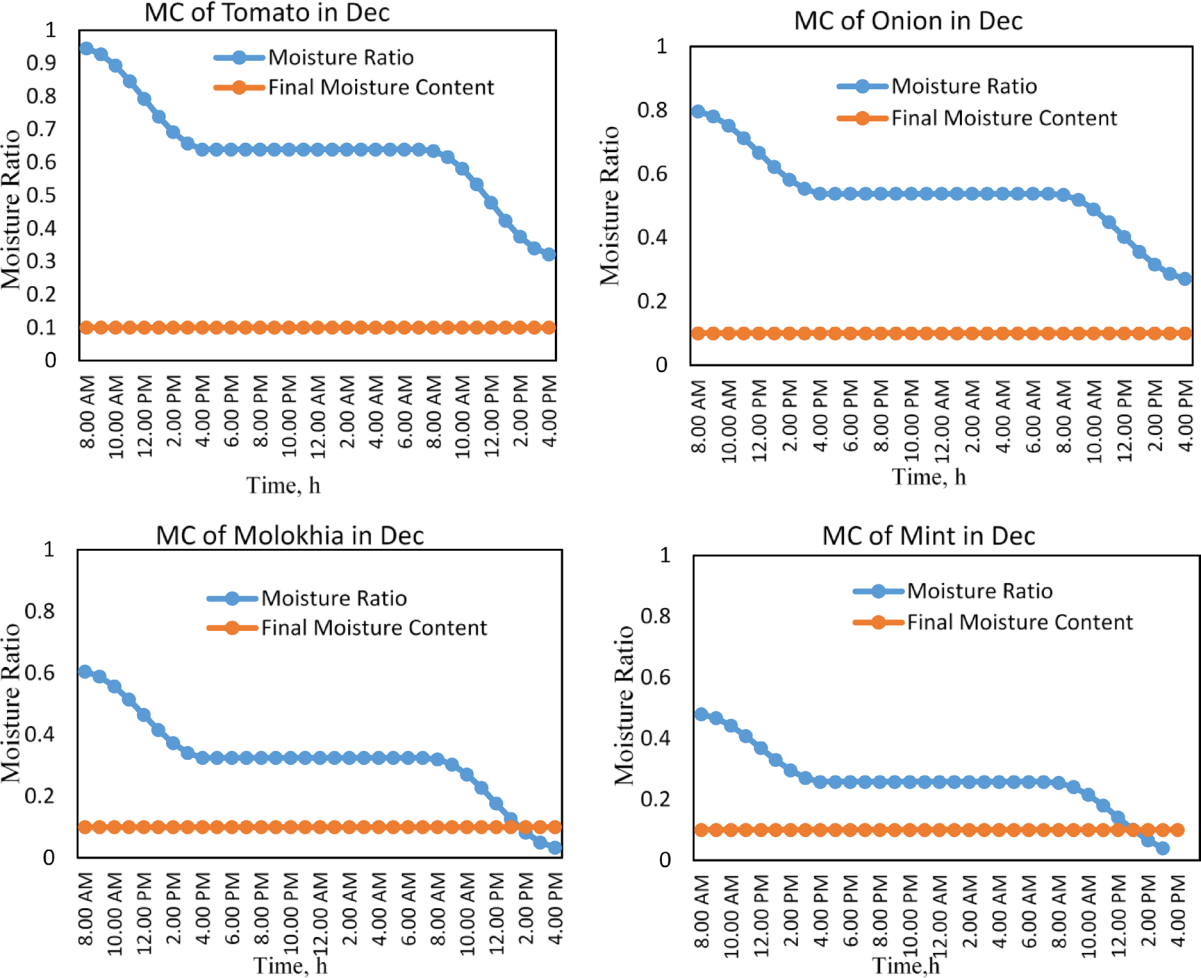
Moisture Content of different products in winter season.

Figure 11 illustrates in the end of the third drying day, the drying process was nearly completed for all tested agricultural products, as the moisture content approached a constant value, indicating that equilibrium moisture content had been reached. For onion samples, the moisture content was approximately 79% at 8:00 AM on the first day and decreased to about 53% by 4:00 PM. The drying process continued gradually during the second day, and by the end of the third day, the moisture content became nearly constant, confirming the completion of the drying process. In the case of Molokhia leaves, a relatively faster drying behavior was observed. The moisture content decreased from approximately 60% at 8:00 AM to about 30% by 4:00 PM on the first day. Only slight reductions were observed during the second day, indicating a faster moisture removal rate compared to onion and tomato due to its thin structure and higher surface-area-to-volume ratio. Similarly, mint leaves exhibited the highest drying rate among the tested products. The moisture content decreased from approximately 47% to 25% within the first drying day, with only minor changes observed on the second day. The drying process was completed by the end of the third day. The observed differences in drying behavior can be attributed to variations in product structure, porosity, and internal moisture diffusion resistance, where leafy vegetables showed faster drying kinetics compared to sliced products such as tomato and onion.

**Fig. 12:**
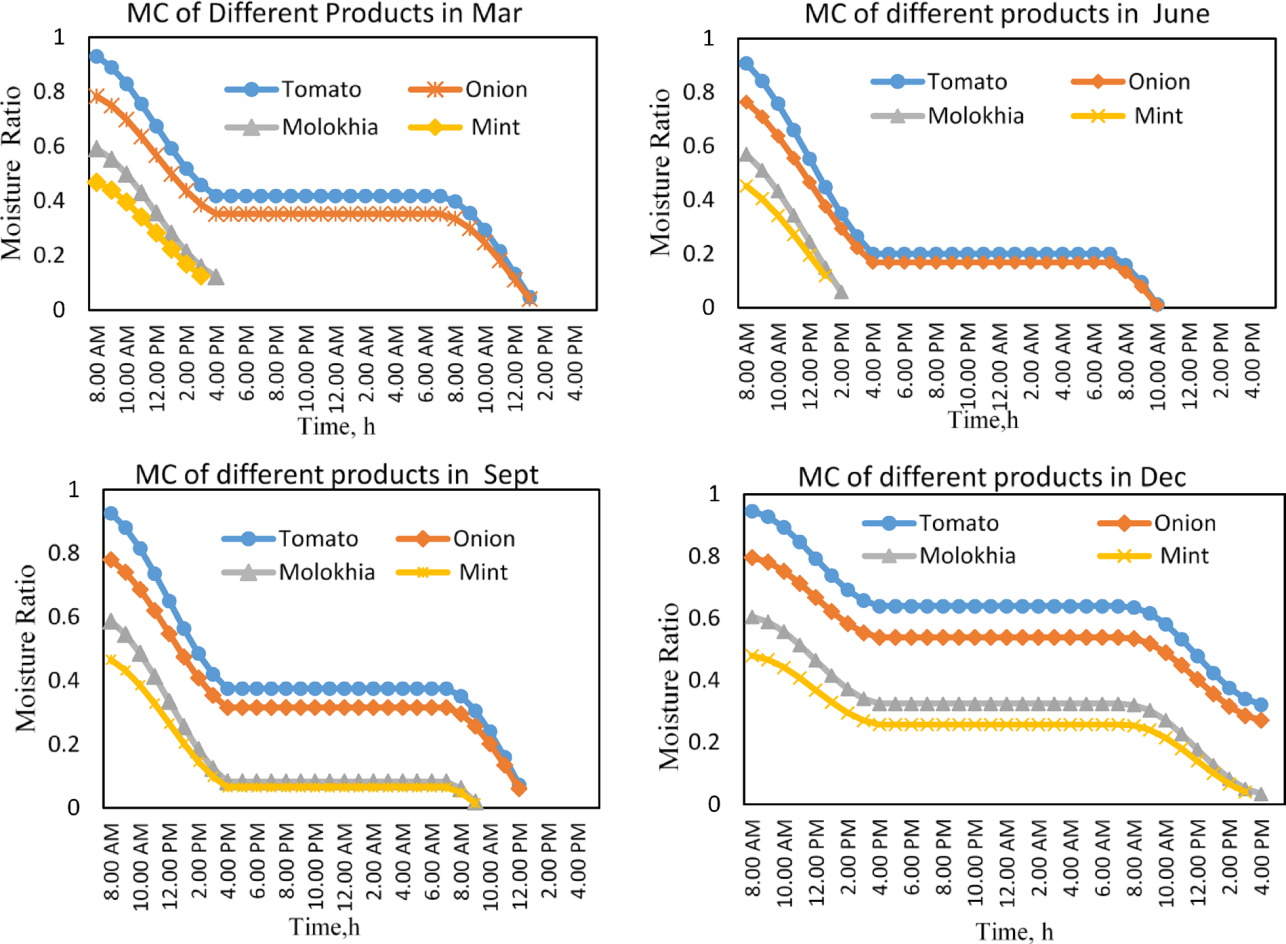
The variation of moisture content of different products versus time at different seasons.

Figure 12 illustrates the variation in moisture content of different agricultural products, namely tomato, onion, Molokhia, and mint, under different seasonal conditions including March, June, September, and December. The results indicate that tomato consistently exhibits the highest moisture content during the drying process, followed by onion and Molokhia, while mint shows the lowest moisture content. This behavior is primarily attributed to differences in the structural composition, initial moisture content, and internal mass transfer resistance of each product. Leafy vegetables such as mint and Molokhia demonstrated faster drying behavior compared to sliced products in March and June, reaching their final moisture content within approximately one drying day. This can be explained by their thin structure, higher surface-area-to-volume ratio, and lower internal resistance to moisture diffusion, which enhance the rate of heat and mass transfer. However, seasonal variations significantly affected the drying performance. In September and particularly in December, the drying time increased due to reduced ambient temperature and lower solar radiation intensity, which decreased the thermal driving force for moisture evaporation. As a result, the products required approximately one full drying day followed by additional hours on the second day to reach equilibrium moisture content. Overall, the observed trends confirm that both product. Characteristics and seasonal environmental conditions play a crucial role in determining the drying kinetics of agricultural products in the solar dryer system.

**Fig. 13:**
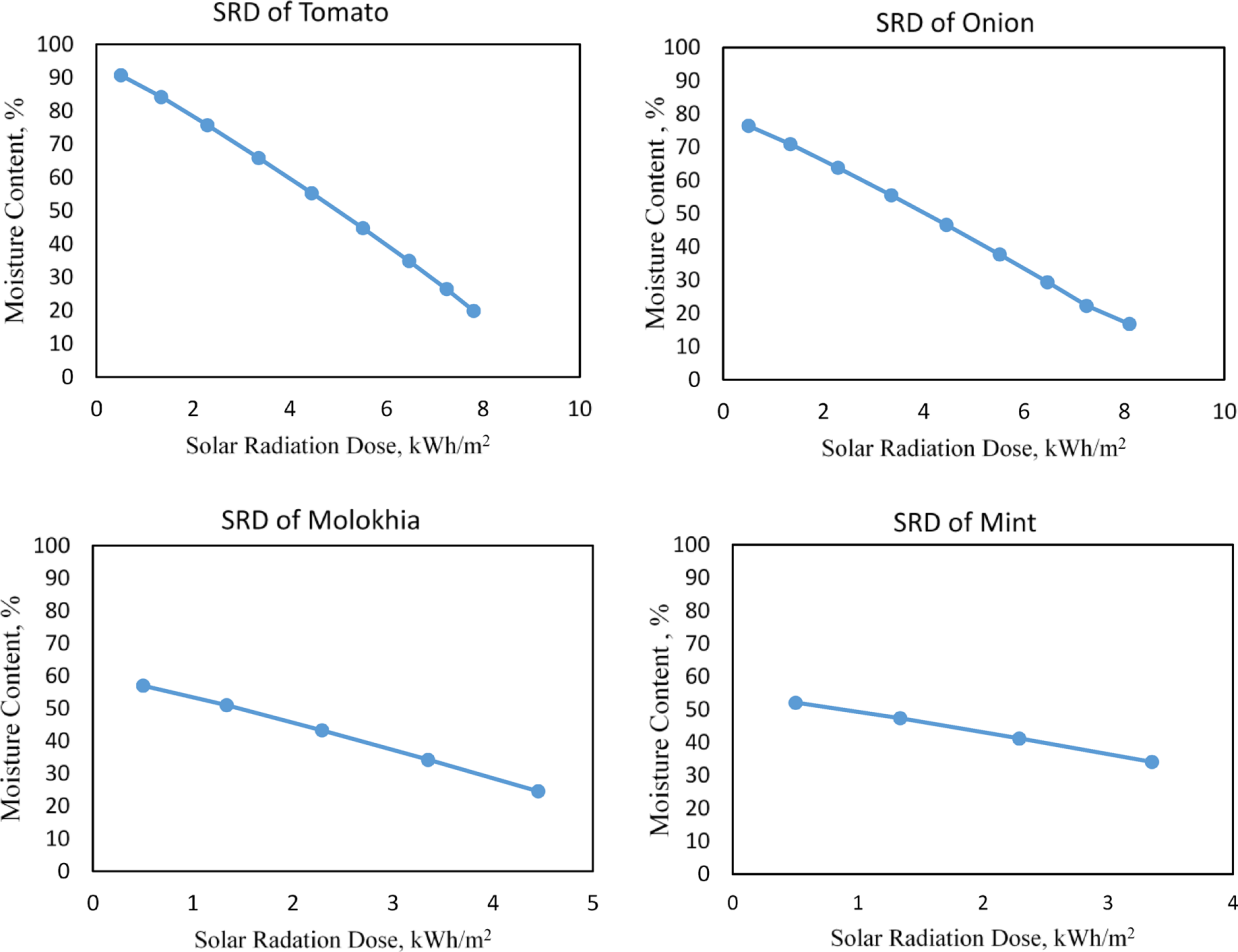
The variation of moisture content versus the solar radiation dose.

Figure 13 illustrates the relationship between moisture content and solar radiation dose (SRD) for tomato, onion, Molokhia, and mint during the drying process. The results show that the total solar radiation energy required for drying varies depending on the type of agricultural product, reflecting differences in their moisture content, structure, and mass transfer resistance. The required solar radiation doses were approximately 7.8 kWh/m² for tomato, 8.1 kWh/m² for onion, 4.2 kWh/m² for Molokhia, and 3.35 kWh/m² for mint. It is observed that leafy vegetables, such as Molokhia and mint, required significantly lower solar radiation doses compared to sliced products. This is attributed to their thin structure and lower internal resistance to moisture diffusion, which enhances the rate of heat and mass transfer during drying. The results indicate that the solar radiation dose required for drying leafy products is lower than the average daily solar radiation in Giza, Egypt (approximately 5.5 kWh/m²/day), which confirms that these products can be effectively dried within a single day under the proposed system. In contrast, tomato and onion require higher solar radiation doses, corresponding to longer drying periods ranging from approximately 1.5 to 3 days, depending on the available solar intensity. The difference between tomato and onion drying behavior can be explained by variations in internal moisture migration pathways, where tomato exhibits relatively faster moisture diffusion compared to onion due to structural differences and tissue porosity. To further evaluate the performance of the solar drying system from an energy perspective, the relationship between solar radiation input and moisture removal can be considered through specific energy indicators. The variation in solar radiation dose (SRD) among different products reflects differences in energy utilization during the drying process. Leafy vegetables such as molokhia Molokhia and mint Mint require lower energy input per unit area due to their thin structure and reduced internal resistance to moisture diffusion, whereas sliced products such as Tomato and Onion require higher energy input due to longer diffusion paths and higher moisture retention. This indicates that product morphology plays a key role in determining the effective energy demand of the drying process, even under similar environmental conditions.

**Fig. 14:**
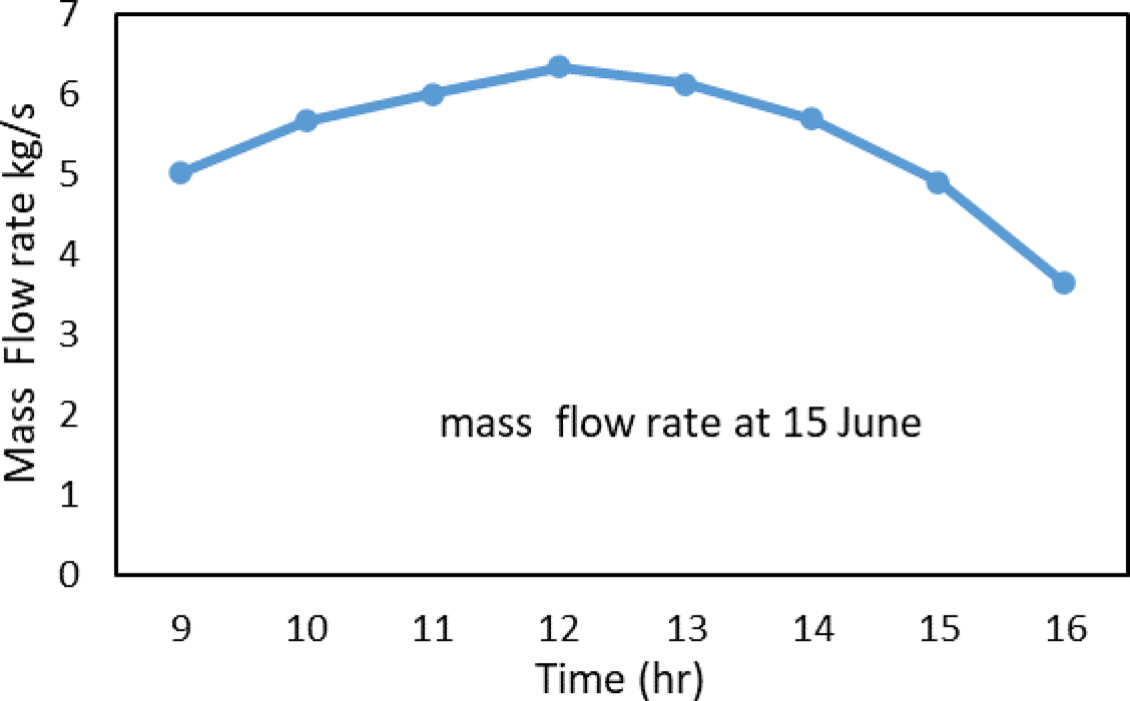
Temporal variation of mass flow rate inside the drying system on 15 June.

Figure 14 shows the air mass flow rate inside the drying chamber was theoretically estimated using the measured air velocity, cross-sectional area, and air density. The results show that the mass flow rate varies between approximately 4.0 and 7.1 kg/s depending on the operating conditions and solar heating intensity. This variation reflects the transient nature of natural convection inside the dryer, which is strongly influenced by temperature differences and buoyancy-driven airflow.

**Fig. 15:**
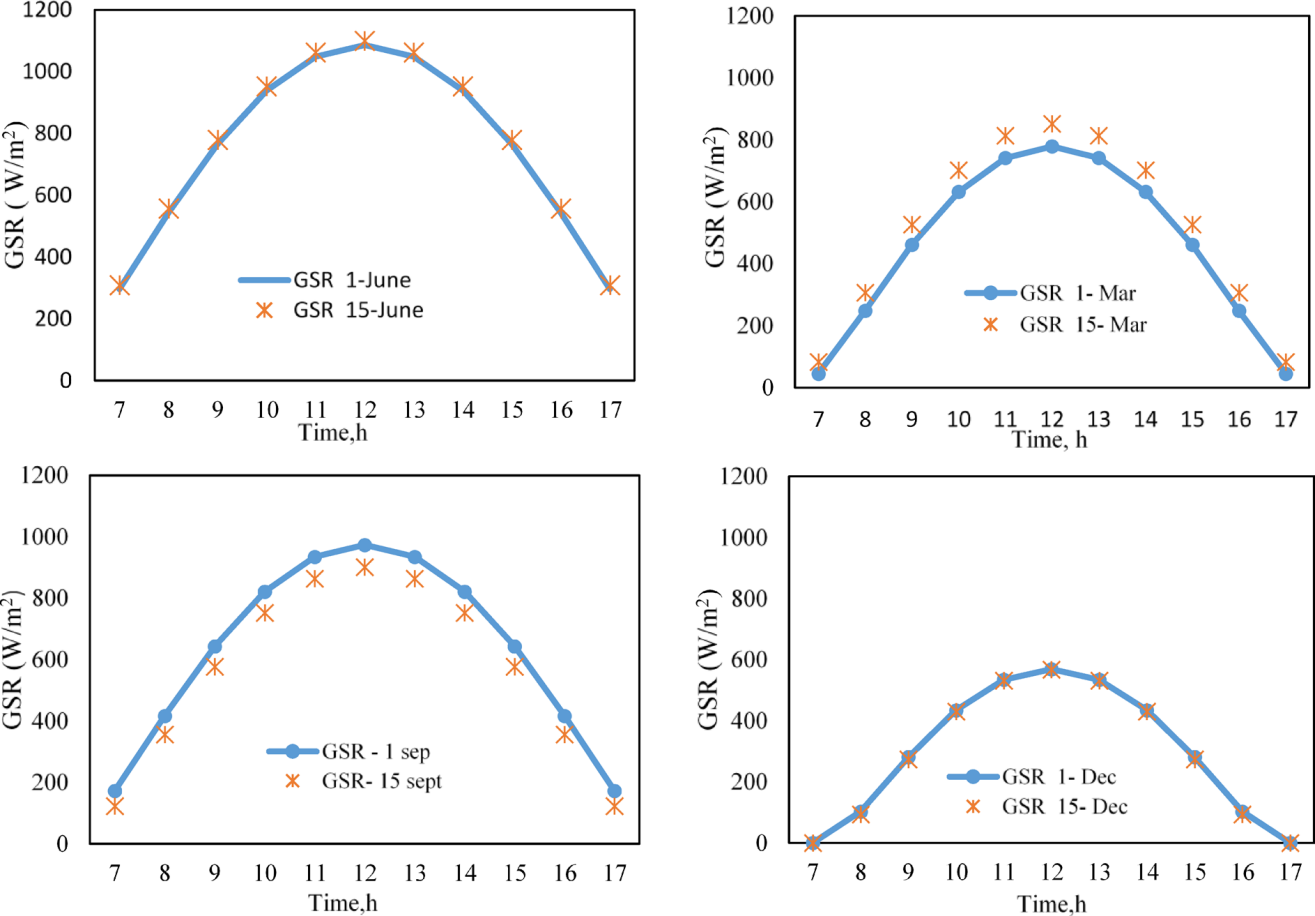
Calculated data of hourly global solar radiation at some days of each month.

The hourly Global solar radiation (GSR) is calculating after taking all the assumptions and affecting parameters into consideration. The calculations are done on daily basis throughout the year. Large No. of solar radiation data is delivered from the simulation program from first January to 31st of December. Figure 15 presents representative solar radiation data over two different selected days for each season, illustrating the annual variation in solar radiation intensity. The results clearly show the typical diurnal solar radiation pattern, where solar intensity increases gradually after sunrise, reaches a maximum value around midday, and decreases progressively toward zero after sunset. This seasonal and daily variation in solar radiation significantly influences the drying performance, as higher radiation levels enhance the thermal energy available for moisture evaporation, thereby accelerating the drying process.

## Statistical validation and error analysis

The model predictions were compared with the experimental measurements using residual analysis. The residuals, defined as the difference between measured and theoretical values, showed both positive and negative deviations across the dataset, indicating that the model does not exhibit a systematic bias and is able to capture the overall trend of the experimental data.

Experimental validation was performed over a wide operating range consisting of multiple data points covering different temperature levels, ensuring a comprehensive assessment of the model performance. To quantify the model accuracy, several statistical indicators were computed. The Mean Absolute Error (MAE) was found to be 2.79, indicating a relatively low average deviation between predicted and measured values. The Root Mean Square Error (RMSE) was calculated as 3.42, reflecting the overall dispersion of the residuals and emphasizing the sensitivity of the model to larger deviations. In addition, the Mean Absolute Percentage Error (MAPE) was determined to be 7.58%, which confirms a high level of agreement between the theoretical model and the experimental results. Overall, the combined statistical and residual analyses demonstrate the robustness, accuracy, and reliability of the proposed model, with randomly distributed residuals around zero and errors within an acceptable range.

The proposed solar drying system demonstrates strong potential for industrial-scale application due to its simple design, low operational cost, and reliance on renewable solar energy. The modular nature of the system allows for straightforward scaling by increasing the collector area and drying chamber capacity, making it suitable for both small scale farmers and medium sized agro processing facilities.

From an application perspective, the system can be integrated into rural agricultural production chains to reduce postharvest losses and improve product preservation without dependence on conventional energy sources. This is particularly relevant for developing regions where access to continuous electrical energy is limited.

However, large scale deployment requires consideration of operational variability due to fluctuating solar radiation and seasonal changes. To enhance reliability, hybrid configurations incorporating auxiliary heating systems or thermal storage units may be considered to ensure consistent drying performance under non-ideal weather conditions. Additionally, proper control of airflow distribution and system insulation becomes increasingly important at larger scales to maintain uniform drying conditions.

Overall, the system provides a feasible and environmentally sustainable solution for agricultural drying applications, with clear pathways for industrial adaptation and integration into existing food processing infrastructure.

## Conclusion

- The results demonstrate a clear seasonal dependency in the solar drying behavior of the investigated agricultural products. Leafy materials such as Molokhia and mint consistently exhibited faster drying rates compared to non leafy vegetables such as tomatoes and onions under all seasonal conditions. This behavior can be attributed to fundamental heat and mass transfer mechanisms, where the higher surface-area-to-volume ratio and thinner structure of leafy products enhance both convective heat transfer and moisture diffusion, resulting in reduced internal resistance to moisture migration.
- Seasonal variations significantly influenced the drying kinetics. During winter (December), the drying time increased notably for all products, reaching up to approximately two days to achieve final moisture content. This slowdown is directly linked to reduce solar radiation intensity, lower ambient temperatures, and increased relative humidity, which collectively decrease the vapor pressure gradient between the product surface and surrounding air, thereby reducing the driving force for moisture evaporation.
- In contrast, more favorable meteorological conditions during spring, summer, and autumn (March, June, and September) enhanced the heat and mass transfer processes, allowing most products particularly leafy vegetables to reach equilibrium moisture content within a single day. The increased solar radiation improves sensible heat gain, which accelerates internal moisture diffusion and surface evaporation rates.
- The observed differences between product categories further confirm that product morphology plays a dominant role in drying kinetics under natural solar conditions. Leafy vegetables, due to their porous structure and low moisture binding strength, facilitate faster internal moisture migration compared to dense tissues found in onion and tomato.
- The obtained results are consistent with previous studies on solar drying systems, which emphasize the strong influence of both product geometry and environmental conditions on drying performance. Similar trends have been reported, where leafy vegetables show significantly shorter drying times compared to high-moisture and dense crops due to enhanced effective diffusivity and reduced internal resistance.
- From an energy perspective, it was observed that molokhia Molokhia and Mint require solar radiation input lower than the average daily solar radiation in Giza, Egypt (≈ 5.5 kWh/m²/day), indicating that complete drying can be achieved within a single day under favorable conditions. In contrast, tomato Tomato and onion Onion require higher solar energy input due to higher moisture content and stronger internal binding of water within the cellular structure. Tomato exhibited relatively faster drying than onion, which is attributed to lower internal resistance to moisture migration.
- Quantitatively, tomato required an average solar radiation dose of approximately 7.8 kWh/m²/day, corresponding to a drying period of 1.5–2 days depending on solar availability. Onion required approximately 8.1 kWh/m²/day, resulting in a drying time of 2 to 3 days under varying conditions.
- Overall, the results highlight the coupled influence of environmental conditions and product specific physical properties on solar drying performance, providing a comprehensive framework for predicting drying behavior under natural conditions.

## Future work

- Integrating thermal energy storage or hybrid heating systems to improve drying stability under fluctuating solar conditions. Further studies are recommended to incorporate advanced airflow control strategies and investigate a wider range of agricultural products. In addition, coupling experimental work with numerical simulations (e.g., CFD modeling) and performing techno economic analysis would provide deeper insight into large-scale feasibility and optimization of system design.
- Including continuous monitoring of relative humidity to allow a more comprehensive analysis of its effect on drying kinetics.
- Evaluating specific energy consumption and comprehensive energy efficiency analysis of the system.

## Data Availability

The data presented in this study is available on request from the corresponding author.
